# Geographic Divisions and Modeling of Virological Data on Seasonal Influenza in the Chinese Mainland during the 2006–2009 Monitoring Years

**DOI:** 10.1371/journal.pone.0058434

**Published:** 2013-03-19

**Authors:** Jingyang Zou, Hua Yang, Hengjian Cui, Yuelong Shu, Peipei Xu, Cuiling Xu, Tao Chen

**Affiliations:** 1 School of Mathematical Sciences, Beijing Normal University, Beijing, China; 2 State Key Laboratory of Remote Sensing Science jointly sponsored by Beijing Normal University and the Institute of Remote Sensing Applications of CAS, School of Geography and Remote Sensing Science, Beijing Normal University, Beijing, China; 3 School of Mathematical Sciences, Capital Normal University, Beijing, China; 4 National Institute for Viral Infectious Disease Control and Prevention, Chinese Center for Disease Control and Prevention, Beijing, China; University of Oxford, Viet Nam

## Abstract

**Background:**

Seasonal influenza epidemics occur annually with bimodality in southern China and unimodality in northern China. Regional differences exist in surveillance data collected by the National Influenza Surveillance Network of the Chinese mainland. Qualitative and quantitative analyses on the spatiotemporal rules of the influenza virus's activities are needed to lay the foundation for the surveillance, prevention and control of seasonal influenza.

**Methods:**

The peak performance analysis and Fourier harmonic extraction methods were used to explore the spatiotemporal characteristics of the seasonal influenza virus activity and to obtain geographic divisions. In the first method, the concept of quality control was introduced and robust estimators were chosen to make the results more convincing. The dominant Fourier harmonics of the provincial time series were extracted in the second method, and the VARiable CLUSter (VARCLUS) procedure was used to variably cluster the extracted results. On the basis of the above geographic division results, three typical districts were selected and corresponding sinusoidal models were applied to fit the time series of the virological data.

**Results:**

The predominant virus during every peak is visible from the bar charts of the virological data. The results of the two methods that were used to obtain the geographic divisions have some consistencies with each other and with the virus activity mechanism. Quantitative models were established for three typical districts: the south1 district, including Guangdong, Guangxi, Jiangxi and Fujian; the south2 district, including Hunan, Hubei, Shanghai, Jiangsu and Zhejiang; and the north district, including the 14 northern provinces except Qinghai. The sinusoidal fitting models showed that the south1 district had strong annual periodicity with strong winter peaks and weak summer peaks. The south2 district had strong semi-annual periodicity with similarly strong summer and winter peaks, and the north district had strong annual periodicity with only winter peaks.

## Introduction

The epidemic of seasonal influenza displays a seasonal pattern as well as the activity of seasonal influenza virus [1]–[5]. Influenza epidemics occur annually with marked winter peaks in most countries and regions in the northern hemisphere, such as the United States, Canada and Europe [6], [7]. However, surveillance in the Chinese mainland has shown a remarkable dual pattern of seasonal influenza: a regular winter pattern for northern China, which is similar to the regions listed above, and a different pattern for southern China. In southern China, both summer and winter peaks exist [8].

In related studies, the main types of seasonal influenza surveillance data used in statistical analyses are usually mortality [9], Influenza-Like Illness (ILI) and virological data [6], [10]–[12]. Alonso et al. (2007) studied the seasonality of influenza throughout Brazil by modeling influenza-related mortality data from 1979 to 2001 for each of the 27 Brazilian states. De-trended time series were analyzed by a Fourier decomposition to describe the amplitude and timing of annual and semiannual epidemic cycles, and the resulting seasonal parameters were compared across latitudes, ranging from the equator (+5°N) to the subtropics (−35°S) [9]. Meijer et al. (2006, 2007 and 2008) conducted research on clinical and virological data on influenza from 33 countries collected by the European Influenza Surveillance Scheme (EISS) to assess influenza activity in Europe during the winters of 2004–2005, 2005–2006 and 2006–2007. In the three articles, the level and the transmission direction of the influenza epidemics were analyzed by the dominant virus subtypes [10]–[12]. The domestic research on ILI and virological data in the Chinese mainland are mostly limited to a certain sentinel hospital or selected provinces; global analyses on the entire country are rare [13]–[14]. Yan Gao et al. (2009) initially analyzed the ILI and virological data of seasonal influenza in the Chinese mainland during the 2006–2009 monitoring years. In that paper, spatiotemporal cluster methods and spatial trend surface methods were used to study the spatiotemporal characteristics of seasonal influenza and to explore its transmission patterns [6].

Most articles using virological data focus on the dominant subtypes and transmission direction, such as the research in [6], [10]–[12]. The study of the spatiotemporal characteristics of seasonal influenza was often conducted using ILI data. Little attention was paid to the regularities of the time series of the total number of detected viruses. Our paper obtains more information from this type of time series.

Considering the current surveillance and research situation in the Chinese mainland, the authors of [6] used conventional geographic divisions to divide the Chinese mainland into northern and southern parts, following the Qinling Mountain range to the west and the Huai River to the east. Compared with the surveillance network in the United States and Europe, the conventional geographic divisions in China are imprecise. In the United States, influenza surveillance was initially conducted in 9 districts, and the number of districts later increased to 10. In Europe, surveillance work is performed at the country level [15]. Our research on geographic divisions and quantitative models verifies and refines the current Chinese geographic divisions, further revealing the spatial differences in influenza virus activity. These divisions are important for the adoption of effective measures for prevention and control according to local conditions.

In general, our work can be separated into two parts: geographic divisions and quantitative modeling on typical districts. The virological data are from the National Influenza Surveillance Network. The period of analysis is from April 2006 to March 2009, and the space covers 31 provinces in the Chinese mainland.

## Materials and Methods

### The National Influenza Surveillance Network

Before 2006, most of the sentinel hospitals in the National Influenza Surveillance Network used the traditional paper method to make records, causing serious missing data problems in the data collection process. Since 2006, the hospitals in the network have reported surveillance data through the Internet, which has triggered a great improvement in data normalization and a decrease in the missing data rate. However, due to the H1N1 influenza pandemic in 2009, a change was made to the statistical criteria: the fever temperature of ILI cases was modified to ‘>37.5°C’ from the original value of ‘>38°C’. In June of 2009, the government launched a project to enlarge the National Influenza Surveillance Network. In this project, the number of sentinel hospitals and influenza laboratories was increased to 556 and 411 from 197 and 63, respectively.

Before it was extended in June 2009, the National Influenza Surveillance Network had been comprised of 63 influenza laboratories and 197 sentinel hospitals across 31 provinces in the Chinese mainland. The sentinel hospitals were chosen taking one province as a unit. The central hospital, the comprehensive hospital and the maternity hospital in the province are of primary importance. The sampling of the sentinel hospitals is heterogeneous in space. For influenza surveillance purposes, the Chinese mainland was divided into northern and southern parts following the Qinling Mountain range to the west and the Huai River to the east. In 13 of the 16 northern provinces, surveillance began from the week of October 1 and ended in the week of March 31 of the following year. In the 3 northern provincial areas of Liaoning, Tianjin and Gansu and in all southern provinces, surveillance was conducted throughout the year. Thus, the analysis, summary and report of these surveillance data are performed separately for the northern and southern regions [16].

For convenience, the period from April to the following March is called a monitoring year in this paper. A database of surveillance information from April 2006 to March 2009, that is, the 2006–2009 monitoring years, was established from the National Influenza Surveillance Network.

### Virological data on influenza activity in the Chinese mainland

The data from the National Influenza Surveillance Network consist of information about ILI cases and virus subtypes. The sentinel hospitals define ILI cases according to the World Health Organization criteria: the sudden onset of a fever (>38°C), a cough or sore throat, and the absence of other diagnoses. The number of ILI cases and the total number of outpatients at the sites (ILI consultation rate) were recorded each day and reported to the National Influenza Surveillance Information System each week. Sentinel hospitals were required to collect 5–15 nasopharyngeal swabs each week from ILI patients who had not taken antiviral drugs and who had a fever (>38°C) for no longer than 3 days. The swabs were sent to the corresponding influenza laboratories for virus isolation and identification; results were reported to the National Influenza Surveillance Information System within 24 hours [8], [17].

The virological data used in our paper refer to information about virus subtypes. The provinces under surveillance throughout the year have 159 weekly data in total, from the 13th week in 2006 (2006-03-27 to 2006-04-02) to the 14th week in 2009(2009-04-09 to 2009-04-12). The remaining provinces, except Tibet (where the data are missing), have only 81 weeks of data. The data include 6 categories: influenza A (H1N1), influenza A (H3N2), influenza A (unsubtyped), subtype B/Yamagata, subtype B/Victoria and influenza B (unsubtyped).

Limited by the sampling number (5–15 samples per week), the virological data on seasonal influenza in a single sentinel hospital are generally scant and have poor regularity. The lack of regularity indicates that the data are better summarized for a larger space scale. Considering that surveillance work is currently conducted at the provincial level, compiling data at the provincial level is not only more theoretically feasible but more meaningful on a practical level.

### Geographic divisions based on influenza virus activity

The data analyzed in the paper are provincial time series of the total number of detected influenza virus. Two methods were used to analyze the influenza virus activity laws of different provinces to obtain geographic divisions.

The first method is called “Peak Performance Analysis”. In this method, the concepts of quality control were introduced to define the “threshold”, “peak period”, “peak value” and “peak width” of the time series. If two provincial time series have similar values of the above four quantities in every monitoring year, the provinces should have similar peak performance. We created corresponding geographic divisions based on this similarity.

Fourier harmonic extraction is the second method. The first step of this method was to extract dominant Fourier harmonics from the provincial time series. Then, we applied the VARiable CLUSter (VARCLUS) procedure in SAS (Statistics Analysis System) software to variable cluster the extracted results and obtained the geographic divisions.

Of note, we found that of the 159 weeks of data in Guizhou province, 142 weeks appeared to be 0. Too many zeros make the provincial time series almost like a straight line without peaks and periodic patterns. Thus, the two methods of ‘Peak Performance Analysis’ and ‘Fourier Harmonic Extraction’ are not suitable to analyze it. Considering the same situation didn't turn up in other provinces, we separated Guizhou into a distinct district. In addition, a preliminary analysis revealed that the three northern provinces with year-round surveillance had low numbers in the months from April to September. Therefore, they were treated in the same way as other northern provinces, that is, only analyzing the data of these three northern provinces in the surveillance periods of other northern provinces.

1. Peak Performance Analysis

From the viewpoint of influenza pandemic laws research, the peak performance of the time series is particularly important. The peak period, peak value and peak width are closely related to the start time of an influenza pandemic, the influenza prevalence and the epidemic duration. The analysis on these descriptive characteristics has important practical significance for the description of the influenza virus activity laws and the understanding of influenza pandemic laws. In our paper, a curve's peak period is defined as the part beyond a certain threshold, while the peak value is defined as the maximum of the peak period.

We used a quality control chart to identify a “peak”. The control chart was invented by Walter A. Shewhart in the 1920s. He understood that data from physical processes typically produce a normal distribution curve. However, observed variation in manufacturing data did not always behave the same way as data in nature. Dr. Shewhart concluded that while every process displays variation, some processes display controlled variation that is natural to the process, while others display uncontrolled variation that is not present in the process causal system at all times.

Similar to manufacturing data, the time series of virological data can be assumed to have a normal distribution if no epidemics occur. In other words, the process is in control. Once peaks occur, the virological process becomes an uncontrolled one with the variations that can be detected by the quality control charts.

Specifying the control limits is one of the critical decisions in designing a control chart. Regardless of the distribution of the quality characteristic, it is standard practice in the United States to determine the control limits as a multiple of the standard deviation (which can be denoted as 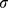) of the statistic plotted on the chart. The multiple usually chosen is 3; hence, 3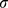 limits are customarily employed on control charts, regardless of the type of chart employed. Correspondingly, 2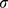 limits are additionally employed as warning limits [18]. If the process is in control, that is, if the quality characteristic is normally distributed, the probabilities of obtaining data outside of the 3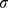 limits and the 2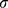 limits are 0.27% and 4.55%, respectively.

**Figure 1:**
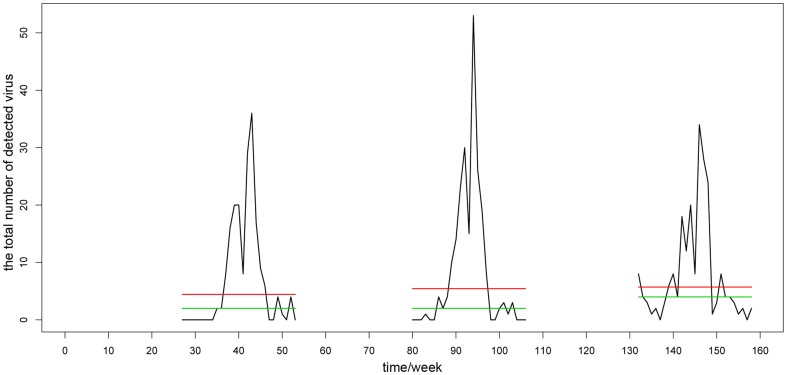
Control chart for the weekly total number of detected viruses (Beijing). It's an example to illustrate the peak performance analysis method. The horizontal ordinate denotes the corresponding number of the 159 monitoring weeks in chronological order. As an example, the 13th week in 2006 is numbered as 1. The black line is the time series of virological data in Beijing. The green lines are the annual medians, and the red lines are the annual 2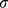 control limits, that is, the thresholds. More information about the results of this method is given in Table S1, S2 in detail.

In our cases, we introduce the warning limits (i.e., 2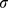 upper control limits) as the threshold to identify peaks. The value of the 2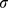 upper control limits is defined by the following equation
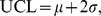
where UCL is an abbreviation for “upper control limits”. For robustness consideration, we utilize the annual (or semi-annual) median to estimate 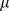 and the corrected annual (or semi-annual) interquartile range (that is, the value of the lower quartile subtracted from the upper quartile), which can be computed by
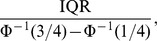
to estimate 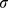, where IQR is annual (or semi-annual) interquartile range, and 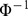 is the inverse function of the standard normal distribution. The median and the IQR are robust location statistics and scale statistics, respectively. The reason for this correction is to ensure the consistency with the true value under normal distribution. As an illustration of the peak performance analysis method, the control chart for the weekly total number of detected viruses of Beijing is given in **Figure 1**.

2. Fourier Harmonics Extraction

To explore the periodicity of the provincial time series of the virological data, a discrete Fourier transformation was considered. The discrete Fourier transformation is a specific kind of discrete transformation generally employed in the field of frequency-domain analysis on time series. In summary, it transforms the original time series to a linear combination of a group of trigonometric functions in discrete frequencies. We analyze the periodicity of the time series by comparing the components corresponding to each trigonometric function. Further details of discrete Fourier transformation can be found in [19].

After the discrete Fourier transformation, dominant Fourier harmonics can be extracted. The superposition results of these harmonics are the basis of the method of variable clusters to obtain geographic divisions. To some extent, the handling procedure can realize the goal of noise reduction. Specifically, the method can strengthen the correlation of the time series that have similar periodicity and weaken the correlation of the time series that have different periodicity. To illustrate the point better, **Table 1** gives the correlation coefficients of the nine southern provinces before and after the Fourier harmonic extraction procedure.

**Table 1:** The correlation coefficients of the nine southern provinces before and after the Fourier harmonic extraction procedure.

	Guangdong	Guangxi	Fujian	Jiangxi	Hunan	Hubei	Shanghai	Zhejiang	Jiangsu
Guangdong	1.00	0.41(0.85)	0.29(0.51)	0.42(0.76)	0.15(0.11)	0.13(0.24)	0.00(−0.13)	0.02(−0.03)	−0.05(−0.12)
Guangxi		1.00	0.47(0.39)	0.45(0.75)	0.25(0.18)	0.12(0.19)	−0.03(−0.16)	0.26(0.04)	0.01(−0.04)
Fujian			1.00	0.45(0.41)	0.21(0.09)	0.17(0.21)	0.03(−0.15)	0.03(−0.06)	0.26(0.00)
Jiangxi				1.00	0.45(0.46)	0.33(0.71)	0.08(0.24)	0.28(0.36)	0.16(0.31)
Hunan					1.00	0.63(0.78)	0.49(0.83)	0.62(0.74)	0.32(0.83)
Hubei						1.00	0.57(0.73)	0.54(0.69)	0.55(0.83)
Shanghai							1.00	0.39(0.67)	0.46(0.81)
Zhejiang								1.00	0.29(0.72)
Jiangsu									1.00

*Note: The values in the parentheses represent the correlation coefficients after the Fourier harmonic extraction procedure.*

From the table, we can see that the correlation coefficients between the first four provinces are larger after the Fourier harmonic extraction procedure. As an example, the correlation coefficient between Guangdong and Jiangxi increases from 0.42 to 0.76. The same thing happens on the data of the last five provinces. On the other hand, the correlation coefficients of the two provinces in different groups decrease, such as the one between Hunan and Fujian. These changes benefit the VARCLUS procedure to do the division more accurately.

Some robust methods were additionally applied in the process of the Fourier harmonic extraction due to the limited data length and the non-robustness of the Fourier transformation. The two reasons could cause some outliers to have a much larger impact on the results of the Fourier analysis than other data. Consequently, the generality would be masked by the peculiarity and contingency, which is not we have expected. To obtain robust results, we replaced these outliers (in our case, the points whose absolute value of standardized residuals was greater than 2) with their extracted results, and then, performed a re-harmonic extraction using the data after above replacement procedure. This kind of replacement is a general method employed in robust statistics (similar treatment can be seen in the robust filter algorithm in [20]). However, too many replacement incidences might prevent the raw data from being sufficiently absorbed, so we limited the instances. Specifically, for the southern provinces, the maximum time of replacements was 3, and for the northern provinces, that was 2. As a result, the proportion of the replaced points in every provincial time series is all below 20%.

**Figure 2:**
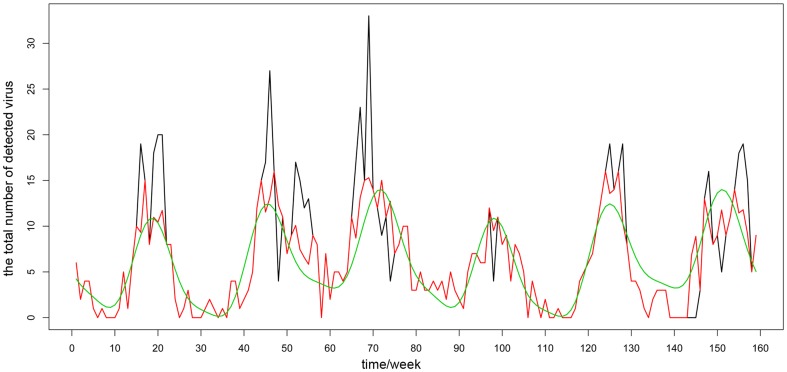
The Fourier harmonic extraction results of Hunan province. It's an example to illustrate the Fourier harmonic extraction method. The horizontal ordinate denotes the corresponding number of the 159 monitoring weeks in chronological order. The black line is the time series of raw data. The red line is the data after the replacement procedure and the green line is the superposition results of the extracted Fourier harmonics. More information about the results of this method is given in Table S3 in detail.

Another important problem was determining the number of harmonics. The criterion was to select the fewest harmonics necessary for a superposition on the foundation of sufficiently preserving the periodicity characteristics of the original time series. Too many harmonics would diminish the significance of “noise reduction”, and the corresponding components were additionally difficult to explain in practice. We employed Fisher's test, which is generally used to test whether there are hidden periodicities of unspecified frequency in time series [19], [21], to solve the problem. The number of harmonics was chosen by the test with p-value<0.01. As an example, **Figure 2** gives the Fourier harmonic extraction results of Hunan province.

The VARCLUS procedure used in our research is a standard procedure in SAS software. It attempts to divide a set of variables into nonoverlapping clusters in such a way that each cluster can be interpreted as essentially unidimensional. For each cluster, PROC VARCLUS computes a component that can be either the first principal component or the centroid component and tries to maximize the sum across clusters of the variation accounted for by the cluster components. PROC VARCLUS is a type of oblique component analysis related to multiple group factor analysis [22]. More details and examples can be found in PROC VARCLUS, SAS Help and Documentation. In the research, we chose the CENTROID option (Centroid components are unweighted averages of the standardized variables), while others are just the default ones. Moreover, the proportion of the total variation that the cluster components can explain should be more than 75%.

### Modeling on influenza virus activity data

To quantify the regularities of seasonal influenza virus activity and to provide a basis for the subsequent modeling of seasonal influenza spread and risk assessment, we applied sinusoidal curves to perform the nonlinear least square fitting and simulate the time series of the total number of detected viruses in typical districts; these typical districts were obtained from the results of the above geographic divisions. There were two reasons for selecting sinusoidal models. First, sinusoidal models are widely used to fit time series with obvious periodicity, as is the case for the virological data. Moreover, each parameter in sinusoidal models has a clear corresponding practical meaning. The double-sinusoidal model in the southern districts is
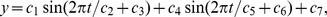
where 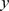 denotes the total number of detected viruses, 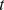 denotes the corresponding number of the 159 monitoring weeks in chronological order, and 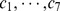 are the parameters to be estimated. To explain, 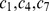 are parameters for peak height, 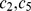 are parameters for cycle and 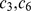 are parameters for phase. The single-sinusoidal model in the northern district is
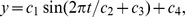
where 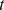 denotes the corresponding number of the 81 monitoring weeks in chronological order. The parameters 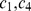 denote peak height, 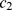 is a parameter for cycle, and 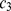 is the parameter for phase.

## Results

### Predominant virus subtypes

**Figure 3:**
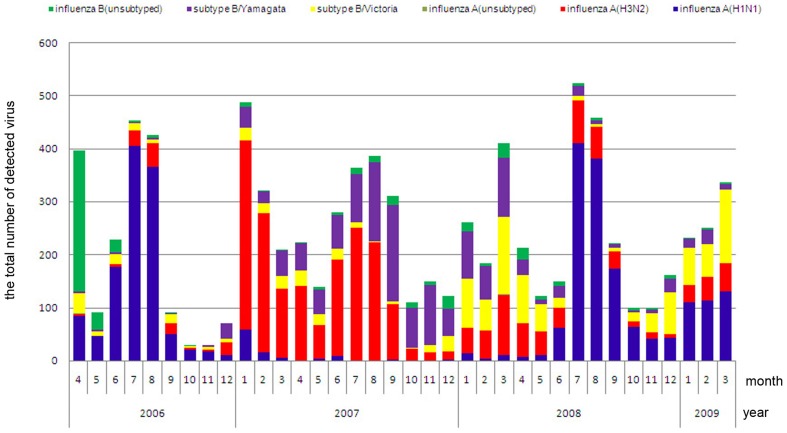
Bar chart of the influenza virus subtypes in the southern area. Different colors represents different influenza subtypes as is listed in the head.

**Figure 4:**
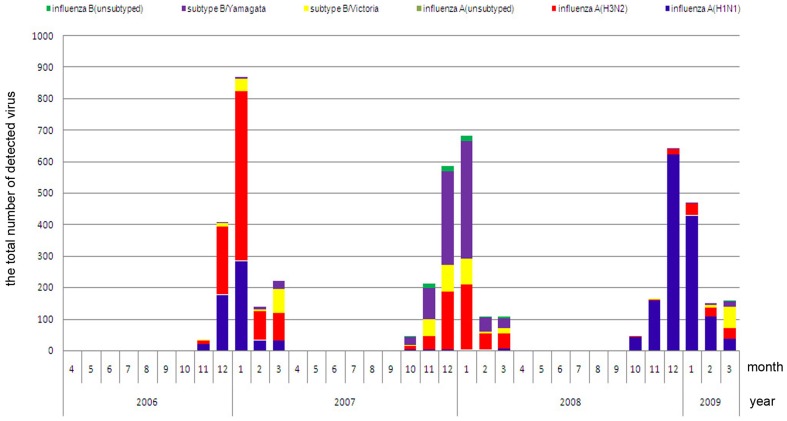
Bar chart of the influenza virus subtypes in the northern area. Different colors represents different influenza subtypes as is listed in the head.

According to the bar charts showing influenza subtypes (**Figure 3** and **Figure 4**), the predominant influenza virus subtypes causing the three peaks in northern area were influenza A(H1N1) and influenza A(H3N2) in winter 2006–2007, influenza A(H3N2) and subtype B/Yamagata in winter 2007–2008, and influenza A(H1N1) in winter 2008–2009. In the southern area, the main active periods of H1N1 were the summers in 2006–2007 and 2008–2009, and the winter in 2008–2009; the active periods of H3N2 were the winter in 2006–2007 and the summer in 2007–2008. Subtype B/Yamagata was active throughout the 2007–2008 monitoring year in the southern area, while Subtype B/Victoria partly caused the 2007–2008 and 2008–2009 winter peaks.

Considering we have only three monitoring years' data, it is difficult to study the activity laws of each influenza virus subtype. Hence, our research focused on the provincial time series of the total number of detected viruses.

### Geographic divisions based on influenza virus activity

1. Results of the Peak Performance Analysis method

**Figure 5:**
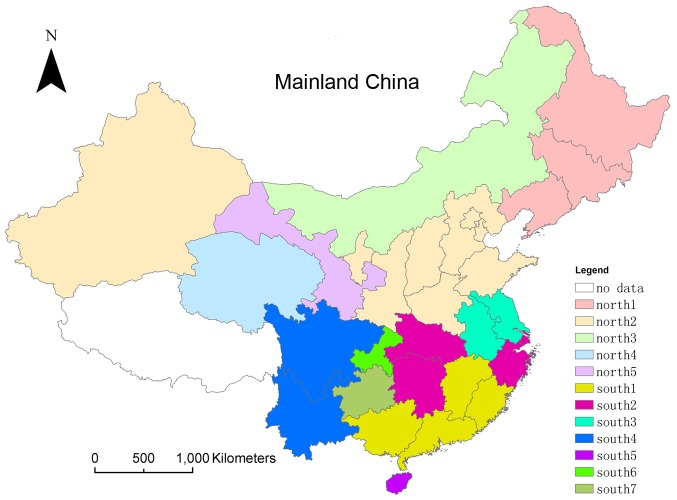
Geographic division results of Mainland China based on the peak performance analysis method. The provinces with the same color belong to the same district.

The peak performance analysis method has an intuitive result as is shown in **Figure 5**. The geographic division results are mainly based on the descriptive characteristics including threshold, peak period, peak value and the corresponding week of peak value, of the provincial time series. The specific results are listed in Table S1, S2.

Guangdong, Guangxi, Fujian and Jiangxi provinces were divided into the south1 district; Hunan, Hubei, Shanghai and Zhejiang provinces into the south2 district; Anhui and Jiangsu provinces into the south3 district; Yunnan and Sichuan provinces into the south4 district; and Hainan, Chongqing and Guizhou provinces, respectively, were placed in the south5-south7 districts. For the northern provinces, Heilongjiang, Jilin and Liaoning provinces were categorized as the north1 district; Beijing, Hebei, Henan, Shandong, Shanxi, Shaanxi, Tianjin, Ningxia and Xinjiang provinces as the north2 district; and Neimeng, Qinghai and Gansu provinces, respectively, as the north3-north5 districts.

All provinces divided into the first three southern districts displayed consistent annual periodicity. The emergence of active time in summer and winter was relatively fixed, where the summer peaks of the south1 district appeared in June–July and the winter peaks in March–April; the summer peaks of the south2 district in July–August and the winter peaks in February–March; and the summer peaks of the south3 district in August and the winter peaks in January–February. Meanwhile, the virological data of Hainan province showed weak overall volatility; the annual periodicity in Yunnan, Sichuan and Chongqing provinces was not obvious. The active virus period of the first two northern districts was December to the following January, however, the north1 district's peak period occurred slightly earlier than the north2 district. Neimeng and Qinghai showed weak periodicity, while Gansu appeared bimodal in both the 2006–2007 and 2007–2008 monitoring year.

2. Results of the Fourier Harmonic Extraction method

The first step of the Fourier harmonic extraction method was to choose the first few harmonics with the maximum amplitude. It is beneficial to obtain data's inherent periodicity. Alternately, the replacement process might lose some information from the raw data. However, we still got favorable geographic division results with above parameters' setting.

**Figure 6:**
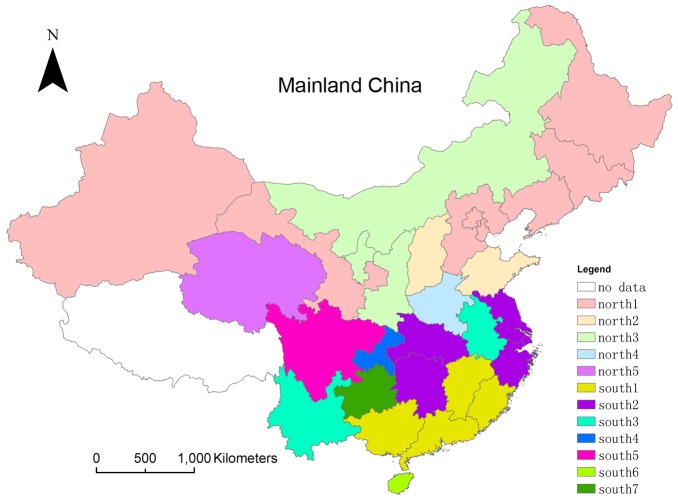
Geographic division results of Mainland China based on the Fourier harmonic extraction method. The provinces with the same color belong to the same district.

The results of the VARCLUS procedure based on the Fourier harmonic extraction are given in **Figure 6**. Guangdong, Guangxi, Fujian and Jiangxi provinces were included in the south1 district; Hunan, Hubei, Zhejiang, Shanghai and Jiangsu provinces in the south2 district; Anhui and Yunnan provinces in the south3 district; and Chongqing, Sichuan, Hainan and Guizhou provinces, respectively, in the south4–south7 districts. Heilongjiang, Jilin, Liaoning, Beijing, Tianjin, Hebei, Xinjiang and Gansu provinces were categorized as the north1 district; Shandong and Shanxi as the north2 district; Shaanxi, Ningxia and Neimeng as the north3 district; and Henan and Qinghai, respectively, as the north4–north5 districts. Details on the significant harmonics which have passed the Fisher's test are given in Table S3.

Analyses of the results were performed. The south1 district was exactly the same across the two methods. The four provinces in this district displayed strong annual periodicity in the harmonic extraction process. The harmonic superposition results had three broad waves, each with two peaks: one high and the other low. The winter peak periods and the summer peak periods were close to each other and the winter peaks were weaker than the summer peaks. Compared with the results of the peak performance analysis, the south2 district incorporated Jiangsu in the Fourier harmonic extraction method. The six provinces in this district displayed semi-annual periodicity, and the harmonic superposition results had six narrow waves, each with a single peak. We can interpret this pattern to mean that the summer and winter peaks were both significant. To Anhui and Yunnan, the semi-annual periodic terms were one of their significant harmonics, while other high-amplitude periodic terms also showed up. The same circumstance also happened on the data of Chongqing. An analysis of the raw data showed that Sichuan experienced a virus-not-active year, the 2006–2007 monitoring year. The periodic analysis on the northern provinces showed that most of the provinces, except Liaoning and Qinghai, had strong annual periodicity. However, the second largest amplitude of the periodic term of Liaoning was still the annual periodicity. The overall performance of northern provinces was quite consistent. We found out that the main reason for Shandong and Shanxi being separated out might be that their peaks turned up a litter earlier each year. Shaanxi, Ningxia and Neimeng demonstrated a strong annual periodicity, but the second and third largest amplitude terms did not significantly decrease. Henan provinces had special circumstances without a significant peak in the 2007–2008 monitoring year. Qinghai showed a 1.5 year periodicity, and the annual periodicity was relatively weak.

### Modeling on influenza virus activity data

According to the above geographic division results and the information obtained in the analytical process, we chose three typical districts to perform sinusoidal fitting. Specifically, they were the south1 district, including Guangdong, Guangxi, Jiangxi and Fujian provinces; the south2 district including Hunan, Hubei, Shanghai, Jiangsu and Zhejiang provinces; and the north district including 14 northern provinces except Qinghai.

**Figure 7:**
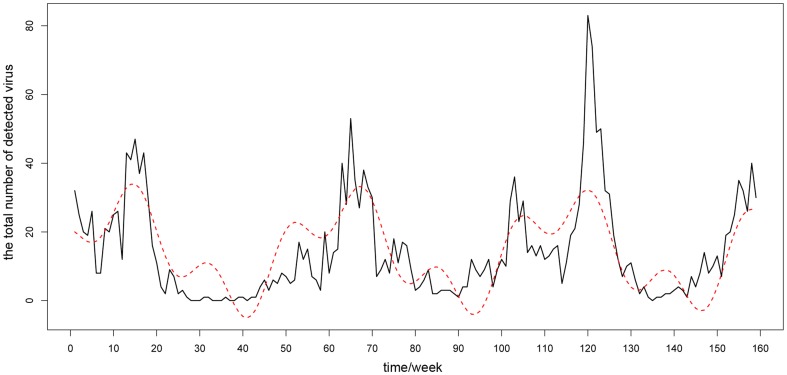
The comparison between raw data and sinusoidal model fit results for the south1 district. The black line is the time series of raw data, while the red line is the sinusoidal fitting curves.

In general, the south1 district showed strong annual periodicity with high summer peaks and slightly lower winter peaks. The south2 district showed strong semi-annual periodicity with significant summer peaks and winter peaks. And, the north district showed single winter peaks. **Figures 7** **,** **8** **, and** **9** give the comparison between raw data and sinusoidal model fit results for the south1 district, the south2 district and the north district, respectively. The coefficient estimates of three regional models are shown in **Table 2** and **Table 3**.

**Figure 8:**
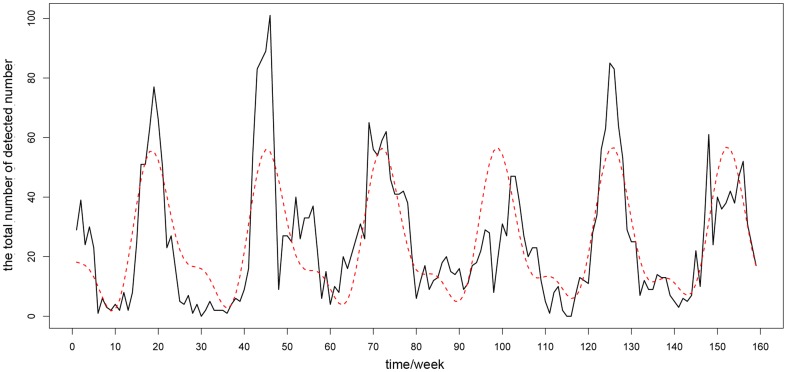
The comparison between raw data and sinusoidal model fit results for the south2 district. The black line is the time series of raw data, while the red line is the sinusoidal fitting curves.

**Figure 9:**
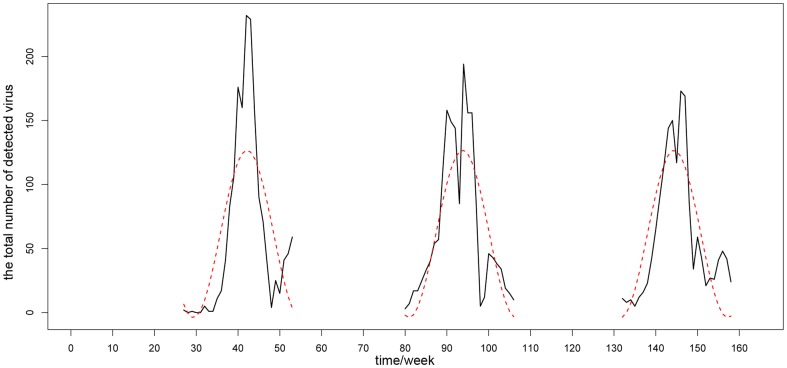
The comparison between raw data and sinusoidal model fit results for north district. The black line is the time series of raw data, while the red line is the sinusoidal fitting curves.

**Table 2:** The estimated coefficients of sinusoidal fitting models for the southern districts.

Parameters for peak height						Parameters for cycle				Parameters for phase			
south1 district			south2 district			south1 district		south2 district		south1 district		south2 district	
*c*_1_	*c*_4_	*c*_7_	*c*_1_	*c*_4_	*c*_7_	*c*_2_	*c*_5_	*c*_2_	*c*_5_	*c*_3_	*c*_6_	*c*_3_	*c*_6_
13.34	7.26	14.32	−22.26	9.64	25.1	51.68	17.64	26.59	13.44	0.14	8.76	0.07	−0.48

**Table 3:** The estimated coefficients of sinusoidal fitting model for the northern district.

Parameters for peak height		Parameter for cycle	Parameter for phase
*c*_1_	*c*_4_	*c*_2_	*c*_3_
−68.07	64.34	25.49	−5.56

The estimated model parameters results give the following information: the south1 district had a strong annual periodicity (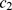 = 51.68 weeks
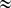
159/3 = 53 weeks); the south2 district had a strong semi-annual periodicity (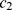 = 26.59 weeks
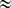
159/6 = 26.5 weeks); and the north district also had a strong annual periodicity (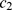 = 25.49 weeks
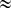
81/3 = 27 weeks). Additionally, for the southern districts, because the amplitude parameter 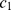 is significantly larger than 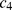, we can believe that the periodicity shown by the corresponding period parameter 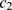 is stronger than the periodicity shown by 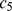. That is the reason for why we pay most attention on 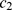 rather than 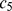.

## Discussion

In the first part of the paper, two methods were applied to create geographic divisions, and both methods offered unique advantages and emphases. On the one hand, the peak performance analysis method focused on obtaining information about the influenza epidemic period, including timing, duration and severity. On the other hand, the Fourier harmonic analysis method mainly studied the periodicity of the provincial time series. The results of the two methods were generally consistent, but the inconsistencies still need to be explored. It is difficult to determine which method yielded more reliable results. To further resolve this question, additional data are needed. However, to establish the quantitative models, we selected three typical districts based on the comparison between the two methods. These districts were described in the second part of the paper.

We introduced the whole method of quality control, including the assumption of normal distribution. The inventor of quality control charts assumed that the natural physical processes are usually normally distributed, which we considered also happens in the circumstance of virological data without epidemics. However, current researches have not provided enough evidence for how this kind of data distributed. Even so, if there is sufficient proof displaying that the data perform another distribution, which is quite different from the normal distribution, this method can also be used with some modifications. In our paper, we chose the warning limits (i.e. 2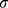 upper control limits) as the threshold to identify peaks and the probability of the obtaining data outside of the limits are 4.55% in normal cases. To non-normal data, it is natural to replace 2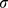 limits with 0.0455 probability limits. The calculation of the 0.0455 probability limits still depends on the assumed distribution. For example, if the distribution is the standard exponential distribution, the corresponding 0.0455 probability limits are actually the 3.1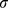 limits, as 3.1 is the 95.45% ( = 1–4.55%) quantile of the standard exponential distribution. More details can be found in [18]. Thus, if we can acquire the real distribution of the virological data, and it's much different from normal distribution, we also can use this method with some modifications. At present, the distribution is commonly assumed as normal.

In the periodicity analysis of the Fourier harmonic extraction, the maximum amplitude periodic terms of several provinces, including Anhui and Qinghai, were the 1.5-year periodic terms. This finding is not easily explained as a result of annual and semi-annual periodicity. We explored potential causal factors, such as “Anhui has a virus-not-active year”. However, the data length is limited, especially for the 1.5-year periodic terms: it only covers two full cycles. Therefore, it is difficult to judge whether this circumstance is a long-term regularity or an accidental phenomenon without additional data and other relevant information. For this reason, we stated the result without drawing conclusions about it. The only certainty is that there are significant differences between the data from these provinces and data from other provinces, so we treated them differently in the construction of the geographic divisions.

Our exploratory analysis of virological data of seasonal influenza is one part of a larger research stream on the epidemic regulations of seasonal influenza in the Chinese mainland. Time series virus data after April, 2009 is acquiring and will be analyzed with these methods, furthermore, the results will be compared to that of these data in the paper. Besides, the analysis of the ILI data is ongoing and has made some advancements. The ILI data from the department of internal medicine have similar spatiotemporal characteristics to the virological data. The results of the two studies can hopefully provide a theoretical basis for the surveillance, prevention and control of seasonal influenza on the Chinese mainland. Research on the relationship between the influenza epidemics and environmental factors is also another issue we will further study in the future.

## Supporting Information

Table S1 **Descriptive characteristic quantities of provincial virological data for peak performance analysis: Southern Provinces.**
(DOC)

Table S2 **Descriptive characteristic quantities of provincial virological data for peak performance analysis: Northern Provinces.**
(DOC)

Table S3 **The amplitudes and corresponding periods of the significant harmonics of each provincial time series.**
(DOC)
