# Supplementary material for: Geographic Divisions and Modeling of Virological Data on Seasonal Influenza in the Chinese Mainland during the 2006–2009 Monitoring Years
Source: PLoS One. 2013 Mar 19;8(3):e58434. doi: 10.1371/journal.pone.0058434 (PMC3602224; doi:10.1371/journal.pone.0058434)
Supplement: Table S1 — Descriptive characteristic quantities of provincial virological data for peak performance analysis: Southern Provinces. (DOC) [file pone.0058434.s001.doc]

Table S1. Descriptive characteristic quantities of provincial virological data for peak performance analysis: Southern Provinces.

|  | **threshold** | **peak period(summer)** | **peak value** | **peak width** | **peak period(winter)** | **peak value** | **peak width** |
| --- | --- | --- | --- | --- | --- | --- | --- |
| ***2006-2007 monitoring year*** | | | | | | | |
| Anhui | 16.83 | 616-617/632 | 24 | 3 | 704-705 | 19 | 2 |
| Chongqing | 11.41 | 625-628 | 19 | 4 | 709 | 12 | 1 |
| Fujian | 5.45 | 613-617/622/625-628 | 12 | 10 | --- | 2(713) | --- |
| Guangdong | 9.41 | 613/620-623/625-627 | 16 | 8 | --- | 5(709) | --- |
| Guangxi | 5.45 | 615/623-631 | 18 | 10 | --- | 3(705) | --- |
| Hainan | 1.48 | 614-616 | 5 | 3 | 709/711-713 | 5 | 5 |
| Hubei | 11.90 | 630-633 | 23 | 4 | 703-707 | 23 | 5 |
| Hunan | 17.34 | 628-628/631-633 | 20 | 4 | 706/713 | 27 | 2 |
| Jiangsu | 13.38 | 614-616/630-631 | 28 | 5 | 702-704 | 38 | 3 |
| Jiangxi | 6.93 | 613/617/626-630 | 20 | 7 | 713 | 9 | 1 |
| Shanghai | 6.93 | 631 | 12 | 1 | 702-707/710 | 32 | 7 |
| Sichuan | 1.48 | 631-633 | 6 | 3 | 646/648-649/701/704/712-713 | 3 | 7 |
| Yunnan | 10.41 | 630/633-634 | 17 | 3 | 706/709 | 14 | 2 |
| Zhejiang | 9.90 | 626-632 | 22 | 5 | 704-707 | 28 | 4 |
| ***2007-2008 monitoring year*** | | | | | | | |
| Anhui | 16.86 | 736-737/739 | 29 | 3 | --- | 14(751) | --- |
| Chongqing | 6.45 | 729-730 | 13 | 2 | 803/807-811/813 | 22 | 7 |
| Fujian | 6.45 | 722-723/725/728/735 | 14 | 5 | 809-812 | 12 | 3 |
| Guangdong | 11.90 | 723/725/728-730 | 19 | 5 | --- | 11(810/812) | --- |
| Guangxi | 5.45 | 723-727/735 | 18 | 6 | --- | 3(811/812) | --- |
| Hainan | 3.97 | 721-722/724-726 | 7 | 5 | --- | 2(802/803) | --- |
| Hubei | 16.86 | 731-732 | 22 | 2 | --- | 13(813) | --- |
| Hunan | 14.90 | 726-729 | 33 | 3 | --- | 12(804) | --- |
| Jiangsu | 12.90 | 730/734-735/737 | 18 | 4 | 809-810 | 19 | 2 |
| Jiangxi | 8.93 | 728-729 | 9 | 2 | 809-810 | 11 | 2 |
| Shanghai | 14.38 | 732-733 | 22 | 2 | 747 | 15 | 1 |
| Sichuan | 5.45 | --- | 4(733) | --- | 802-803/808/810-812 | 19 | 6 |
| Yunnan | 6.93 | 716/736 | 8 | 2 | --- | 6(804/807) | --- |
| Zhejiang | 4.45 | 714-717/733/737-738 | 11 | 7 | --- | 4(808) | --- |
| ***2008-2009 monitoring year*** | | | | | | | |
| Anhui | 1.48 | 832-834/837-838/840-841 | 9 | 7 | 846/901/909 | 5 | 3 |
| Chongqing | 18.34 | --- | 16(815) | - | 850-901 | 26 | 4 |
| Fujian | 7.45 | 832-833 | 12 | 2 | 908-909/913-914 | 12 | 4 |
| Guangdong | 20.31 | 826-830 | 70 | 5 | 913 | 21 | 1 |
| Guangxi | 5.45 | 828-830 | 11 | 3 | 907/910 | 6 | 2 |
| Hainan | 2.97 | 828-830 | 5 | 3 | --- | 2 | --- |
| Hubei | 11.90 | 830-833 | 24 | 4 | 909/911 | 14 | 2 |
| Hunan | 18.83 | 832/835 | 19 | 2 | 911 | 19 | 1 |
| Jiangsu | 11.41 | 831-834/837 | 35 | 5 | 903 | 18 | 1 |
| Jiangxi | 11.90 | --- | 11(831) | --- | --- | 9(909/910) | --- |
| Shanghai | 15.86 | 832 | 16 | 1 | --- | 15(905/906) | --- |
| Sichuan | 5.45 | 816/824/832-833 | 9 | 4 | 911-912 | 8 | 2 |
| Yunnan | 4.97 | 814/829-831/833/836-837/841/843 | 9 | 9 | 851/909-910 | 6 | 3 |
| Zhejiang | 6.93 | 830-832/834-835 | 13 | 5 | 903-904/908/911 | 13 | 4 |

*Note: The three-digit numbers in the table represent the weeks in the following way: the first one represents the year and the last two represent the numbered week. For example， “614” means the 14^th^ week in 2006.*
