# Supplementary material for: Geographic Divisions and Modeling of Virological Data on Seasonal Influenza in the Chinese Mainland during the 2006–2009 Monitoring Years
Source: PLoS One. 2013 Mar 19;8(3):e58434. doi: 10.1371/journal.pone.0058434 (PMC3602224; doi:10.1371/journal.pone.0058434)
Supplement: Table S2 — Descriptive characteristic quantities of provincial virological data for peak performance analysis: Northern Provinces. (DOC) [file pone.0058434.s002.doc]

Table S2. Descriptive characteristic quantities of provincial virological data for peak performance analysis: Northern Provinces.

|  | **threshold** | **peak period** | **peak value** | **peak width** |
| --- | --- | --- | --- | --- |
| ***2006-2007 monitoring year*** | | | | |
| Beijing | 14.60 | 650-652/702-704 | 36 | 6 |
| Gansu | 34.65 | 652-703 | 43 | 4 |
| Hebei | 3.71 | 651-704/706 | 28 | 7 |
| Heilongjiang | 7.41 | 649-652/702-703 | 21 | 6 |
| Henan | 1.48 | 652/702/704-706/709 | 12 | 6 |
| Jilin | 12.12 | 649-650/652-702 | 28 | 5 |
| Liaoning | 6.93 | 651-704 | 15 | 6 |
| Neimeng | 6.67 | 652-703/712-713 | 21 | 6 |
| Ningxia | 0 | 651-703 | 12 | 5 |
| Qinghai | 0 | 705-706/710-713 | 33 | 6 |
| Shandong | 14.34 | 703-707 | 30 | 5 |
| Shaanxi | 4.45 | 701-704 | 11 | 4 |
| Shanxi | 6.67 | 702-705/712-713 | 35 | 6 |
| Tianjin | 11.64 | 701-702/704 | 33 | 3 |
| Xinjiang | 0.74 | 649/651-701/703-704/711 | 10 | 7 |
| ***2007-2008 monitoring year*** | | | | |
| Beijing | 19.79 | 751-802 | 53 | 5 |
| Gansu | 9.15 | 802-803/807 | 16 | 3 |
| Hebei | 6.93 | 750/801-803 | 15 | 4 |
| Heilongjiang | 14.34 | 749-750/752/801 | 21 | 4 |
| Henan | 4.22 | 745/749 | 7 | 2 |
| Jilin | 27.24 | 750 | 30 | 1 |
| Liaoning | 10.15 | 749-751/803 | 24 | 4 |
| Neimeng | 1.48 | 748-752/802 | 11 | 6 |
| Ningxia | 4.71 | 801 | 13 | 1 |
| Qinghai | 0.74 | 743/748-753 | 19 | 7 |
| Shandong | 22.79 | 802-803 | 42 | 2 |
| Shaanxi | 1.48 | 744/746-747/750/752/801 | 8 | 6 |
| Shanxi | 14.6 | 753-802 | 17 | 3 |
| Tianjin | 22.57 | - | 22(751) | - |
| Xinjiang | 3.97 | 750-751/753-801/803 | 8 | 5 |
| ***2008-2009 monitoring year*** | | | | |
| Beijing | 12.9 | 849/851/901-903 | 34 | 5 |
| Gansu | 16.83 | 850/910/912 | 26 | 3 |
| Hebei | 3.97 | 849/902 | 5 | 2 |
| Heilongjiang | 10.41 | 902-903/905 | 22 | 3 |
| Henan | 6.45 | 850-903 | 12 | 6 |
| Jilin | 13.86 | 847/850-851/902 | 22 | 4 |
| Liaoning | 7.67 | 902/905/911 | 17 | 3 |
| Neimeng | 0 | 846-851 | 15 | 6 |
| Ningxia | 11.38 | 848-851 | 21 | 4 |
| Qinghai | 1.48 | 847-901 | 18 | 7 |
| Shandong | 10.9 | 849/851-902 | 27 | 5 |
| Shaanxi | 6.93 | 848/901 | 25 | 2 |
| Shanxi | 9.41 | 848-852 | 24 | 5 |
| Tianjin | 12.15 | 851-903 | 20 | 5 |
| Xinjiang | 3.97 | 851-902 | 12 | 4 |

*Note: The three-digit numbers in the table represent the weeks in the following way: the first one represents the year and the last two represent the numbered week. For example, “650” means the 50^th^ week in 2006.*
