# Supplementary material for: Geographic Divisions and Modeling of Virological Data on Seasonal Influenza in the Chinese Mainland during the 2006–2009 Monitoring Years
Source: PLoS One. 2013 Mar 19;8(3):e58434. doi: 10.1371/journal.pone.0058434 (PMC3602224; doi:10.1371/journal.pone.0058434)
Supplement: Table S3 — The amplitudes and corresponding periods of the significant harmonics of each provincial time series. (DOC) [file pone.0058434.s003.doc]

Table S3. The amplitudes and corresponding periods of the significant harmonics of each provincial time series.

| ***Southern Provinces*** | | | | | | |
| --- | --- | --- | --- | --- | --- | --- |
| Hainan | 15.7(53.0) | 7.2(6.6) | 6.2(9.9) |  |  |  |
| Guangdong | 1178.6(53.0) | 357.2(17.7) | 187(26.5) | 170.5(159.0) | 170.3(39.7) | 127.9(13.3) |
| Guangxi | 276.6(53.0) | 134.9(26.5) | 60.5(17.7) | 36.8(13.2) |  |  |
| Jiangxi | 175.5(53.0) | 116.9(17.7) | 97.6(26.5) |  |  |  |
| Fujian | 161.8(53.0) | 116(79.5) | 51.3(31.8) | 50.2(159.0) |  |  |
| Hunan | 1093.8(26.5) | 130.9(79.5) | 65.3(13.2) |  |  |  |
| Hubei | 873.2(26.5) | 192.2(17.7) | 146.5(53.0) | 123.9(13.2) |  |  |
| Shanghai | 833.7(26.5) | 209.4(13.3) | 70.8(53.0) | 51.8(159.0) | 46.1(19.9) |  |
| Jiangsu | 347.1(26.5) | 97.3(17.7) | 87(13.2) |  |  |  |
| Zhejiang | 341.9(26.5) | 85.3(159.0) | 83.1(31.8) | 54.7(13.3) | 37.8(14.5) |  |
| Anhui | 169.8(79.5) | 135.0(159.0) | 110.3(26.5) | 99.4(17.7) | 71.2(39.7) |  |
| Yunnan | 84.4(26.5) | 33.1(39.8) |  |  |  |  |
| Sichuan | 43.3(159.0) | 23.8(26.5) | 12.9(10.6) |  |  |  |
| Chongqin | 160.3(26.5) | 137.6(159.0) | 103(31.8) |  |  |  |
| ***Northern Provinces*** | | | | | | |
| Heilongjiang | 712.7(27.0) | 148(13.5) | 117.9(20.2) | 70.0(11.6) |  |  |
| Jilin | 1433.1(27.0) | 295.1(13.5) |  |  |  |  |
| Liaoning | 131.3(20.2) | 106.7(27.0) |  |  |  |  |
| Beijing | 2220.7(27.0) | 628.3(13.5) |  |  |  |  |
| Tianjin | 874.7(27.0) | 157.3(13.5) | 90.1(81.0) | 67.4(9.0) | 55.3(16.2) |  |
| Hebei | 134.9(27.0) | 71.5(20.2) | 62.3(81.0) | 34.8(13.5) | 19.8(11.6) |  |
| Henan | 94.9(27.0) | 73.5(40.5) | 64.2(16.2) |  |  |  |
| Shandong | 781.5(27.0) | 207.4(81.0) | 166.2(11.6) | 132.8(20.2) |  |  |
| Shanxi | 417.3(27.0) | 180.1(20.2) | 136.1(11.6) | 83.9(81.0) | 50.4(8.1) | 42.5(13.5) |
| Shaanxi | 41.9(27.0) | 19.7(40.5) | 11.7(16.2) |  |  |  |
| Ningxia | 90.5(27.0) | 87.7(16.2) | 73.7(81.0) | 55.2(40.5) | 26(20.2) | 18.9(13.5) |
| Gansu | 420.1(27.0) | 376.9(13.5) | 371.9(16.2) | 296.6(81.0) |  |  |
| Xinjiang | 96.5(27.0) | 49.8(13.5) | 14(9.0) |  |  |  |
| Neimeng | 118.1(27.0) | 71.2(13.5) | 55.3(11.6) | 52(10.1) |  |  |
| Qinghai | 43.4(40.5) | 36.5(20.2) | 32.3(13.5) |  |  |  |

*Note: The values out of the parentheses are the amplitudes of the significant harmonics of each provincial time series, while the ones in the parentheses are the corresponding periods, whose unit is week.*
